# Effects of modified-Paleo and moderate-carbohydrate diets on body composition, serum levels of hepatokines and adipocytokines, and flow cytometric analysis of endothelial microparticles in adults with metabolic syndrome: a study protocol for a randomized clinical trial

**DOI:** 10.1186/s13063-021-05612-y

**Published:** 2021-09-30

**Authors:** Farnoosh Shemirani, Akbar Fotouhi, Kurosh Djafarian, Leila Azadbakht, Nima Rezaei, Maryam Mahmoudi

**Affiliations:** 1 Department of Cellular and Molecular Nutrition, School of Nutritional Science and Dietetics, Tehran University of Medical Sciences, Tehran, 14155-6447 Iran; 2 Department of Epidemiology and Biostatistics, School of Public Health, Tehran University of Medical Sciences, Tehran, Iran; 3 Department of Clinical Nutrition, School of Nutritional Science and Dietetics, Tehran University of Medical Sciences, Tehran, Iran; 4 Department of Community Nutrition, School of Nutritional Science and Dietetics, Tehran University of Medical Sciences, Tehran, Iran; 5 Diabetes Research Center, Endocrinology and Metabolism Clinical Sciences Institute, Tehran University of Medical Sciences, Tehran, Iran; 6 Department of Community Nutrition, School of Nutrition and Food Science, Isfahan University of Medical Sciences, Isfahan, Iran; 7 Research Center for Immunodeficiencies, Pediatrics Center of Excellence, Children’s Medical Center, Tehran University of Medical Sciences, Tehran, Iran

**Keywords:** Randomized control trial, Metabolic syndrome, Low-carbohydrate diet

## Abstract

**Background:**

Metabolic syndrome is a combination of metabolic risk factors causing a pathological condition that increases the risk of non-communicable diseases, such as diabetes and cardiovascular diseases. A variety of dietary approaches have been examined to halt this rapid trend; however, the effects of modified-Paleo diet and medium-carbohydrate diet on inflammation, adipokines, hepatokines, and the profile of endothelial microparticles in individuals with metabolic syndrome have not been investigated in detail. The present study is designed to examine the effect of modified-Paleo and moderate-carbohydrate diet with two delivery modes: “fixed diet plan” vs “calorie counting” on weight, body composition, serum levels of some hepatokines and adipocytokines, and flow cytometric analysis of endothelial microparticles in adults with metabolic syndrome.

**Methods:**

Eighty metabolic syndrome patients will be recruited in this study. They will be randomly allocated to one of the following 4 groups: (1) receiving a modified-Paleo diet with calorie counting, (2) receiving a modified-Paleo diet with a fixed diet plan, (3) receiving a medium-carbohydrate diet with calorie counting, and (4) receiving a medium-carbohydrate diet with a fixed diet plan for 10 weeks. Weight, height, waist circumference, and body composition will be assessed at the study baseline and at the end of the trial. Serum insulin, asprosin, chemerin, FGF-21, CTRP-1, PYY, ghrelin, plasma EMPs (CD31+/CD42b− and CD144+/CD42b−), lipid profile, glycemic indices, hs-CRP, leptin, vitamin C, creatinine and satiety, hunger, fullness, and desire to eat (via visual analog scales) will be measured at the study baseline and at the end of the trial. Insulin resistance and insulin sensitivity will be determined using the HOMA-IR and QUICKI equations.

**Discussion:**

To the best of our knowledge, this is the first randomized controlled trial that will determine the effect of modified-Paleo and moderate-carbohydrate diet on weight, body composition, serum levels of some hepatokines and adipocytokines, and the profile of EMPs in adults with metabolic syndrome. Moreover, the effects of different diet delivery modes, including “fixed diet plan” and “calorie counting” will also be analyzed. The results of this trial can provide clinical witnesses on the effectiveness of carbohydrate-restricted diets in ameliorating metabolic status and prevent the development of chronic diseases.

**Trial registration:**

Iranian Registry of Clinical Trials IRCT2016121925267N4. Registered on 26 July 2017

**Supplementary Information:**

The online version contains supplementary material available at 10.1186/s13063-021-05612-y.

## Background

Metabolic syndrome is defined as a set of inter-related risk factors including abdominal obesity, hypertension, impaired glucose tolerance, and dyslipidemia. While each of the underlying disorders of metabolic syndrome intensifies the risk of various non-communicable diseases [1], their co-occurrence increases the risk of further complications and mortality [2]. The prevalence of this disorder varies between 14 and 32% globally. Based on the evidence, this syndrome occurs among one-third of adults in Iran [3]. A nutritional transition may be the reason for this high rate of prevalence in Iran which may be resulted from a shift from a traditional diet towards a Western-style diet [4, 5].

Among several attributable risk factors to this public health issue according to almost all of the proposed definitions [6], obesity and insulin resistance are seemed to be the cornerstones of most of the cases of metabolic syndrome [7]. Accordingly, lifestyle alteration such as dietary modifications is still considered the mainstay of treatment to induce weight loss and ameliorate symptoms of metabolic syndrome upon the emergence of its consequences like CVD [8]. Yet, no ideal dietary strategy is consistently superior to others for the general population.

The composition of macronutrients plays a pivotal role in diet recommendations. Conventional low-fat diets have been the most usual dietary strategy for weight reduction for many years, although low-carbohydrate diets with continued success are receiving more attention and popularity in recent years [9]. Despite the fact that low-carbohydrate diets are still controversial, they still are effective with minimum risk and acceptable compliance. A new evaluation on a low-carbohydrate diet is necessary because of several side effects of anti-obesity medications and unacceptable approaches regarding low-fat and energy-restricted diets. One important concern in the field of low-carbohydrate diets is the definitions for this diet.

As mentioned earlier, metabolic syndrome is a chronic and progressive clustering of synergistic metabolic risk factors, which makes the individual more prone to develop especially cardiovascular diseases, approximately twice more than those not having it. Although the particular mechanism of metabolic syndrome pathogenesis has not been understood in detail, researches have shown that biological mediators, known as adipocytokines, produced by adipose tissue contribute to the development of metabolic syndrome [10].

Moreover, the liver plays a role in regulating whole-body metabolism and energy homeostasis via liver-derived proteins known as hepatokines. Hence, hepatokines, besides being valuable predictive biomarkers, could be considered as potential targets for the treatment of cardio-metabolic disorders [11].

Microparticles are cell membrane-shedded fragments, usually expressed by cells during cellular stress and cell activation. Microparticles have a role in the pathogenesis of inflammation [12], cardiovascular diseases [13], and metabolic syndrome [14] and are biomarkers for assessing endothelial function in metabolic syndrome [15].

As carbohydrate-restricted diets have shown to influence inflammation and body composition [16], it is hypothesized that they might affect the level of adipo- and hepatokines and also the level of endothelial microparticles in patients with metabolic syndrome. This factorial design randomized clinical trial is therefore designed to examine the effect of modified-Paleo and moderate-carbohydrate diets with two delivery modes (“fixed diet plan” and “calorie counting”) on weight, body composition, serum levels of some hepatokines and adipocytokines, and flow cytometric analysis of endothelial microparticles in adults with metabolic syndrome.

## Methods/design

This randomized single-blind clinical trial with a factorial design will be conducted in Tehran, Iran. All participants will be asked to complete and sign the written informed consent form before enrollment. The study has already been approved by the bioethics committee of Tehran University of Medical Sciences (No. IR.TUMS.VCR.REC.1396.2046).

### Study population

The current RCT is a community-based participatory research. Participants will be recruited through a well-known online advertising application rather than relying on one health care center or hospital. This was done with the aim of reflecting the diversity of the population and reaching more generalizable conclusions.

### Inclusion criteria

In this study, we will recruit 20–60-years-old patients with metabolic syndrome, at least 1–2 weeks prior to enrollment. Diagnosis of metabolic syndrome will be made based on NCEP-ATPIII [17]. Patients will be recruited if they had at least 3 out of 5 of the abovementioned factors.

### Exclusion criteria

Individuals who are smokers, those consuming alcohol, are pregnant or lactating, or decided to get pregnant during the next 3 months will not be included. Moreover, individuals with some pathologic conditions including cancer, any acute or chronic liver failure, and liver transplantation; suffering from diabetes, heart failure, renal failure, inflammatory disease, and chronic gastrointestinal diseases; usual consumption of corticosteroids and non-steroidal anti-inflammatory drugs (NSAIDs); hormone replacement therapy (HRT); or those taking high dosage of estrogen, insulin, usual receiving of herbals, antioxidant, and multivitamin/mineral supplements during the last 3 months will not be included.

### Study design

**Fig. 1:**
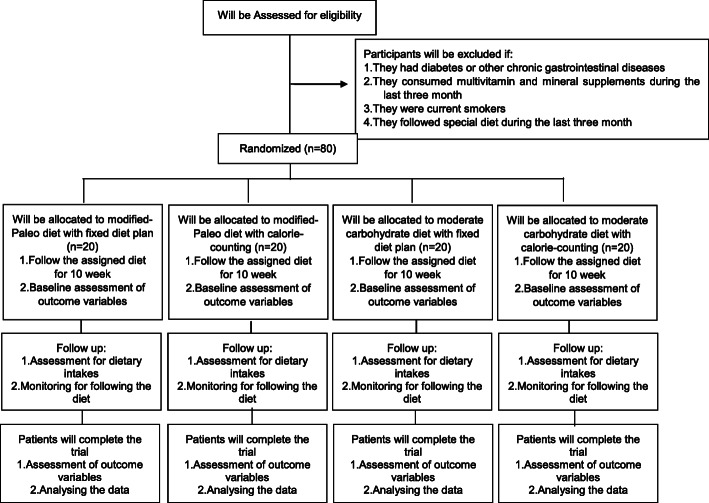
Flow diagram of the study procedure

At the initial phase, 100 patients with metabolic syndrome will be screened based on the inclusion criteria mentioned above. Subjects recruited through online advertisements and social media and those who meet the criteria will be included and are randomly assigned in one of the following 4 groups: (1) those receiving a modified-Paleo diet with calorie counting, (2) receiving a modified-Paleo diet with a fixed diet plan, (3) receiving a medium-carbohydrate diet with calorie counting, and (4) receiving a medium-carbohydrate diet with a fixed diet plan. The principal investigator (FSH) will recruit eligible participants and obtain a consent form. In the consent form, participants will be informed of the study interventions, duration, and contact details of the research team in case of any question. Participants will be told that all health services are free of charge, and they can withdraw their consent at any time of the study. Participants will be told that their participation is on a voluntary basis, and they are required to sign the consent form prior to any data collection. Before allocating participants into the four intervention groups, anthropometric indices including weight, height, and waist circumference (WC) will be measured, and body mass index (BMI) will be calculated. Body composition will be assessed by bioelectric impedance analysis (BIA) according to the instrument instructions. Individual questionnaires including socio-demographic characteristics, past medical, drug and diet history, and validated International Physical Activity Questionnaire (IPAQ) will be also completed for each patient through a comprehensive face-to-face interview. Each individual will be given a booklet consisting of a pre-designed diet program and a series of recipes, meal plans for breakfast, lunch, dinner, and snacks according to the given diet. Those receiving the fixed diet plan method will be provided with special measuring cups with definite colors for each food group. Subjective appetite rating will be assessed by visual analog scales (VAS) to evaluate satiety, hunger, fullness, and desire to eat. To fulfill this, a VAS questionnaire will be given to each participant to fill them every 2 weeks at home. Finally, to perform biochemical measurements, 10-ml overnight fasting venous blood samples will be collected from each participant, and to isolate circulating EMPs from platelet-poor plasma (PPP), 5 ml of venous citrated blood will be obtained from each patient. All personal information of the participants will be confidential in the whole study. Outcome assessors will be blinded to the intervention assignments in the current study. A diagram of the study design is shown in Fig. 1.

### Randomization

Eighty patients will be enrolled in the whole project. We will use a balanced block randomization approach with a block size of 4. The list of randomization will be generated by RALLOC do file of STATA by the study statistician (AF). For concealment, based on this list, a series of randomization envelopes will be prepared. The principal investigator (FSH) will open the recruitment envelopes sequentially and assign participants to interventions.

### Study outcomes

In the present study, we have multiple primary outcomes due to the nature of the dietary interventions as seen in many other randomized clinical trials [18, 19]. A single outcome may be insufficient to fully describe the effects of an intervention on a complex disease, namely metabolic syndrome. Weight, body composition, and CTRP6 (as an inflammatory marker) are the main primary outcomes in the current study. Sample size calculation has been estimated on the body weight changes as the largest number achieved in each group. Hence, our primary endpoints will be the mean difference in the changes from baseline to 10-week follow-up in body composition component (weight and body fat mass) and CTRP6 concentration between the intervention groups. Study success will be declared if either one of the primary endpoints will be statistically significant in favor of the experimental treatment.

The main secondary outcomes would be lipid profile, glycemic indices, serum insulin, leptin, and plasma EMPs (CD31+/CD42b− and CD144+/CD42b−). Satiety, hunger, fullness and desire to eat [20], inflammatory factors (chemerin, FGF-21, Asprosin), PYY, ghrelin, and creatinine are exploratory outcomes.

### Sample size

We calculated the required sample size based on data from a previous study [21]. Sample size calculation was performed with the G*Power V.3.1.9.2 software [22], using the *F* test between the factors with four groups (modified-Paleo diet with calorie counting, modified-Paleo diet with a fixed diet plan, medium-carbohydrate diet with calorie counting, and medium-carbohydrate diet with fixed diet plan) and two evaluation sessions (before and after intervention). According to the mean difference in the change in body weight (11 kg) and standard deviation of 15 (by assuming equal standard deviation in groups) [21], the calculated effect size *f* is 0.39. We considered an *α* error probability of 0.05 and a power (1-*β* error probability) of 0.80. We take the correlation among repeated measures as 0.6. The resulting total sample size is 60, meaning 15 in each intervention group. Allowing for a maximum dropout rate of 30%, the final number of subjects in each treatment group has been set to 20 participants. The study is registered on the Iranian Registry of Clinical Trials website (http://www.irct.ir, identifier: IRCT2016121925267N4). This study was reported based on the recommended checklists for clinical trials [23, 24] (Additional file 1).

### Intervention

The participants will be randomly allocated to one of the four aforementioned groups. Accordingly, each participant will be trained in a pre-designed low- or medium-carbohydrate dietetic regimen during a 1-h face-to-face conversation. Everyone will receive a diet plan based on their weight and their daily calorie needs. In the present study, the modified-Paleo diet is defined as 20% carbohydrate, 35% protein, and 45% fat, and the moderate-carbohydrate diet is defined as 40% carbohydrate, 30% protein, and 30% fat. The cooking tips for some of the foods in modified-Paleo menus will be trained via short video clips, and possible questions will be answered. For those receiving a fixed-diet plan, the specified “measuring cups” for each food group and the supplementary information in the booklets will be completely explained.

### Compliance

To determine the adherence to the intervention, individuals will be asked to record their consumption of the main and between-meals food in a checklist given by the investigators every 4 weeks besides the baseline food records. On the whole, six dietary records will be completed by participants during the study (two work days and one holiday, at baseline and after 10 weeks). All records will be immediately revised in order to find possible weaknesses and resolve them. The Nutritionist IV software (First Databank, San Bruno, CA, USA) which is modified for Iranian foods will be used for the evaluation of the dietary records, and the nutrient intake of each participant will be analyzed accordingly. To increase compliance and avoid forgetting the principles of the diets, participants will receive messages on their cell phones and phone calls from the investigators. In addition, serum vitamin C, which is an indicator for high consumption of fruits and vegetables will be examined.

### Intervention safety

No serious side effects have been reported following carbohydrate-restricted diets. To examine the possible side effects that might arise from taking high-protein diets, serum concentrations of creatinine will be quantified at the study baseline and at the end of the trial.

### Outcome measurements

**Fig. 2:**
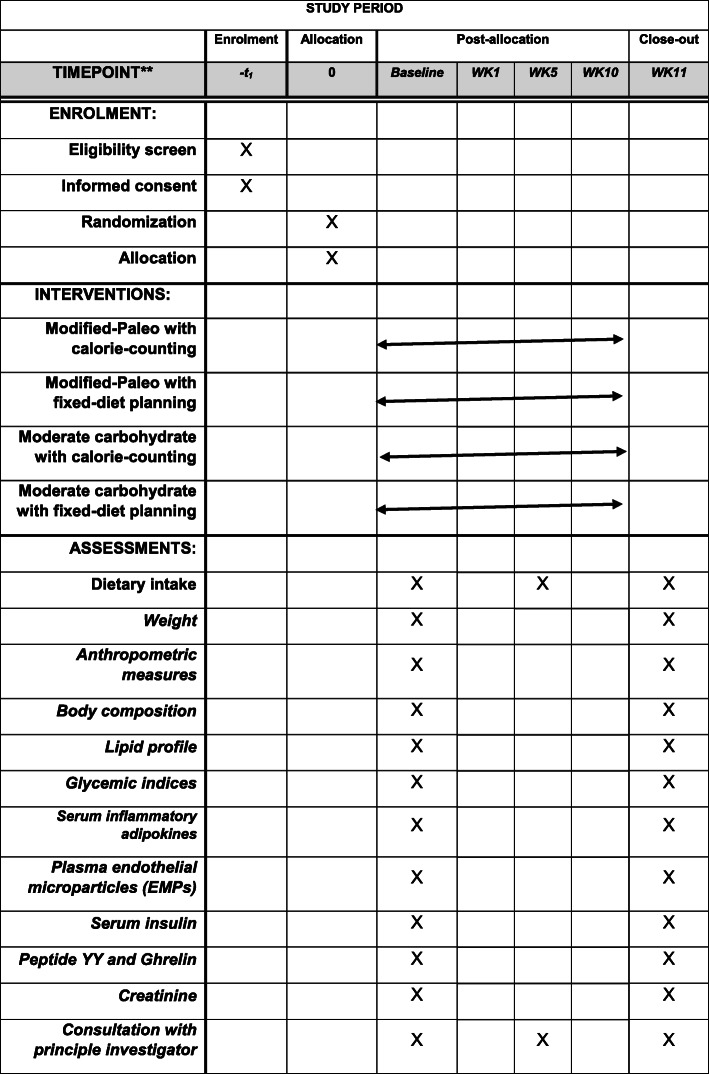
The time point of enrollment, interventions, and assessments. The “X” refers to what has been done in a specific period of time. EMPs, endothelial ,microparticles; WK, week

The time points for the assessments of the outcome measures are demonstrated in Fig. 2. All outcome measures will be assessed at the study baseline and at the end of 10 weeks (WK10). Dietary intakes will be assessed at the study baseline, in the middle of the study (WK5), and at the end of 10 weeks (WK10).

### Physical activity assessment

To assess the level of physical activity of each participant, the International Physical Activity Questionnaire is applied, based on the published guidelines [25]. The levels of physical activity will be classified into low, moderate, and high based on the IPAQ criteria and will be expressed as metabolic equivalents (METs) in minutes per week (MET-min/week).

### Anthropometric assessment

Evaluation of anthropometric parameters, including body weight, height, WC, and BMI will be done at the study baseline and end of the trial. Body weight will be measured after a 12-h overnight fasting, without shoes with minimal clothing to the nearest 100 g accuracy, using a weighing calibrated scale (Seca, Hamburg, Germany). Height will be measured by mounted tape, without shoes and at a standing position near to the wall to the nearest 0.1 cm accuracy using a stadiometer (Seca, Hamburg, Germany).

### Body composition assessment

Evaluation of the body composition will be performed via multi-frequency bioelectric impedance analysis (InBody770, Korea) in order to estimate the changes in body fat mass (FM), percentage of fat mass (PFM), skeletal muscle mass (SMM), and visceral fat (VF) during the study. For more accuracy, the patients will be told to stay hydrated and avoid tea, coffee, and alcohol consumption and physical activity 8 h before the test.

### Blood sampling, biochemical, and flow cytometric measurements of EMPs

In a 12-h fasting state, a 10-ml venous blood sample will be taken from each participant at the beginning and the end of the study. After centrifuging for 15 min (3000 rpm), the serum samples will be separated and simultaneously stored at − 80 °C until analysis. Serum lipid profiles (TC, HDL, LDL, and TG) and blood glucose levels will be measured by the enzymatic colorimetric method using commercial kits. Serum insulin, asprosin, chemerin, FGF-21, CTRP-1, PYY, and ghrelin will be measured using an enzyme-linked immunosorbent assay (ELISA) kit. Insulin resistance and insulin sensitivity will be measured by the HOMA-IR and QUICKI formulae, respectively.

$$ \mathrm{QUICKI}=1/\left(\log\ \left(\mathrm{fasting}\ \mathrm{insulin}\ \upmu \mathrm{U}/\mathrm{ml}\right)+\log\ \left(\mathrm{fasting}\ \mathrm{glucose}\ \mathrm{mg}/\mathrm{dl}\right)\right) $$

$$ \mathrm{HOMA}-\mathrm{IR}=\left(\mathrm{FBI}\ \left(\mathrm{mU}/\mathrm{l}\right)\times \mathrm{FBS}\ \left(\mathrm{mmol}/\mathrm{l}\right)\right)/22.5 $$

Flow cytometric assessment of endothelial-derived microparticles will be performed according to the study of Mikirova et al. [26]. In order to quantify the level of circulating EMPs, 5–10 ml blood will be collected in tubes containing EDTA. The plasma will be processed within 30 min to 1 h after blood collection by two-step centrifugation. Microparticles in the platelet-poor plasma samples will be quantified by flow cytometry as described in previous studies. Briefly, 50 μl of platelet-poor plasma will be incubated with 5 μl of antibodies: anti CD31, CD42b, and CD144, all from Biolegend (San Diego, CA, USA). After staining, the plasma will be diluted by 350 μl of PBS with 10 mmol citrate; 50 μl of 5 μm AccuCount standard beads (Spherotech, Libertyville, IL) will be added to the sample before analysis to allow calculating of MP values. Samples will be analyzed on a flow cytometer (Quanta, Beckman Coulter) with the relevant FlowJo software, and the final data will be expressed as an absolute count of microparticle/μl.

### Confidentiality

All participant’s information will be stored anonymously following TUMS rules. To respect study participant’s privacy and ensure confidentiality, participants will be identified only by ID numbers in all records and when conducting lab tests by lab staff. All identifying characteristics of participants will be stored in a secure database separate from study data collected during the course of the research. All collected data during different study phases will be kept strictly confidential on password-protected computers in the research team’s office which is accessible only by the study staff. Double data entry and regular recruitment procedures will be employed to enhance data quality during data collection. After the trial is complete, anonymized data will be available to other researchers for conducting a meta-analysis from the corresponding author on reasonable request.

### Statistical analysis

Statistical analyses of all data will be performed using STATA version 12.0 (Stata Corp LP, College Station, TX). The intention-to-treat approach will be used for data analysis in order to handle non-adherence. Data will be expressed as mean (SD). The one-sample Kolmogorov–Smirnov test will be used to verify the normality. If a variable distribution is not normal, the log transformation will be applied. An analysis of variance (ANOVA)/analysis of covariance (ANCOVA) model will be used to compare the values among the four intervention groups. Analysis of variance (ANOVA) and post hoc Bonferroni correction will be used for multiple comparison adjustment. Since we have 4 intervention groups (1, modified-Paleo diet with calorie counting; 2, modified-Paleo diet with a fixed diet plan; 3, medium-carbohydrate diet with calorie counting; and 4 medium-carbohydrate diet with a fixed diet plan), we will have six possible pairwise comparisons across the groups (1 with 2, 1 with 3, 1 with 4, 2 with 3, 2 with 4, 3 with 4). By these primary comparisons, we will be able to compare the effects of diets (low vs moderate carbohydrate) and delivery modes (calorie counting vs fixed diet plan) on primary outcomes. Further, there are two modules, by which confidence intervals can also be adjusted along with *p*-value for multiple comparisons. “Pwmean” and “pwcompare” modules allow for performing all pairwise comparisons using Tukey, Bonferroni, or Dunnett. An analysis of covariance (ANCOVA) will be applied to adjust the effects of confounding and baseline variables. *p* < 0.05 (two-sided) will be considered as statistically significant. Besides the null hypothesis significance testing (NHST), appropriate effect size and its 95% confidence interval will also be analyzed to provide more reasonable justification about results. Cohen’s *d* effect size, measured as the mean difference in change divided by the pooled standard deviation of the change, will be reported along with its 95% CI. Cohen’s *d* is defined as standardized mean difference (SMD) and is classified as small (*d* = 0.2), medium (*d* = 0.5), or large (*d* = 0.8) [27]. Multiple imputation will be used to correct missing data. The SAMPL guideline will be used for statistical analysis in this study [28].

### Interim analyses

No interim analysis or stopping rules was anticipated in the current study due to the nature of the interventions. Indeed, no adverse effect or intractable problem was reported regarding dietary approaches with mild to moderate carbohydrate restriction so far. Further, by having weekly contacts with participants, any minor complaint will be handled by making slight changes in the dietary program such as adding or omitting a special food item.

### Plans to give access to the full protocol, participant-level data, and statistical code

The full study protocol is accessible via IRCT.ir (IRCT2016121925267N4). The datasets analyzed during the current study are available from the corresponding author on reasonable request.

### Oversight and monitoring

The lead study coordinators will be MM and KDJ, who conceived the study design and will be in charge of supervising the project. Indeed, MM and KDJ are the main part of the Trial Steering Committee (TSC) along with the vice dean for research and study advisor (LA), statistician and study advisor (AF), study physician (NR), and principal investigator (FSH). The committee will meet every month in order to oversee conduct and progress, overcome probable financial or technical difficulties, and develop plans to meet the projects’ schedules. Due to the low-risk nature of the trial, a separate Data Monitoring Committee is not necessary. The coordinating center is the clinical nutrition and biochemistry lab at Tehran University of Medical Sciences, in which blood sampling, serum collection, storage, and all laboratory tests will be performed. Lab technicians are responsible for providing ELISA kits, antibodies, laboratorial equipment, and needed products. The principal investigator (FSH) takes the responsibility for coordinating visits for identifying potential recruits and taking consent. The Data Management Team will consist of the principal investigator (FSH) and project supervisors (MM and KDJ).

### Frequency and plans for auditing trial conduct

No planned trial auditing has been considered currently. Similarly, no Data Monitoring Committee was considered due to the low-risk nature of dietary interventions. Besides, dietary interventions have been investigated and approved by the Ethics Committee during a strict procedure prior to the beginning of the trial. However, the principal investigator (FSH) is responsible to inform the Trial Steering Committee and Ethics Committee of any unforeseen risks throughout the trial period. The Trial Steering Committee will meet every month in order to oversee conduct and progress, overcome probable financial or technical difficulties, and develop plans to meet the projects’ schedules.

### Plans for communicating important protocol amendments to relevant parties (e.g., trial participants, ethical committees)

Implementing major amendments to the protocol which may affect the conduct of the study or patient safety and benefit will be communicated to the supervisors, investigators, Trial Steering Committee, and any participants affected. Any deviation from the protocol will be fully documented using a breach report form. The principal investigator (FSH) makes updates or edits to the study protocol published on the Iranian Registry for Clinical Trials (IRCT.ir).

### Dissemination

The results of the current trial will be disseminated through interactive workshops with stakeholders, links with policymakers, conference presentations, and a series of papers in open-access peer-reviewed journals.

## Discussion

Metabolic syndrome is one of the most important non-communicable diseases worldwide, which imposes a substantial burden on the health system. Obesity as the result of changing dietary habits is the leading cause of this metabolic disorder which gradually ends in various comorbidities like diabetes and cardiovascular diseases [29]. A superior dietary regimen has not yet been proposed decisively to fight obesity. Carbohydrate-restricted diets, despite lacking a precise definition, have been gaining much attention due to being extensively used for weight reduction and weight control in the past decades [30]. Moreover, it has been shown that a chronic low-grade inflammation is a link between obesity, metabolic syndrome, and type 2 diabetes [31]. Adipose tissue and liver could be sites of inflammation in conditions like obesity, via producing biological mediators with pro- or anti-inflammatory functions. Besides, the circulating pattern of endothelial-derived microparticles could be a valuable prognostic and diagnostic marker in patients with metabolic syndrome, being prone to develop cardiovascular diseases and diabetes [15, 32].

Due to the complex interaction between underlying causes of metabolic syndrome and the complications caused by it, a safe and efficacious dietary approach with a practical delivery mode in real life could successfully halt the rapidly increasing trend of metabolic syndrome prevalence.

Thus, the effects of low- and medium-carbohydrate diets on fat mass, visceral fat, and the level of adipokines, hepatokines, and EMPs in adults with metabolic syndrome will be studied in this trial. The results of the current study could shed light on introducing an appropriate dietary approach to facilitate weight reduction and improve the components of metabolic syndrome.

## Trial status

The current protocol is version 2, dated 23 November 2020. The recruitment has been begun on 22 December 2019. Because of the COVID-19 pandemic, we anticipate a recruitment completion date of 21 January 2021. Any changes to the protocol will be notified to all relevant parties and updated on the Iranian Registry of Clinical Trials (IRCT.ir).

## Supplementary Information

**Additional file 1.** SPIRIT 2013 Checklist: Recommended items to address in a clinical trial protocol and related documents*.

## Data Availability

Any data required to support the protocol can be supplied on request.

## Abbreviations

MetS: Metabolic syndrome
NCD: Non-communicable diseases
CVD: Cardiovascular diseases
EMPs: Endothelial microparticles
VAS: Visual analog scales
FGF-21: Fibroblast growth factor 21
CTRP-1: C1q/TNF-α–related protein 1
PYY: Peptide YY
hs-CRP: High-sensitivity C-reactive protein
HOMA-IR: Homeostatic Model Assessment for Insulin Resistance
QUICKI: Quantitative insulin-sensitivity check index
NCEP: National Cholesterol Education Program
ATPIII: Adult Treatment Panel III
NAFLD: Non-alcoholic fatty liver disease
MPs: Microparticles
CONSORT: Consolidated Standards of Reporting Trials
SPIRIT: Standard Protocol Items: Recommendations for Interventional Trials
SAMPL: Statistical Analyses and Methods in the Published Literature
NSAIDs: Non-steroidal anti-inflammatory drugs
HRT: Hormone replacement therapy
WC: Waist circumference
BMI: Body mass index
BIA: Bioelectric impedance analysis
IPAQ: International Physical Activity Questionnaire
TC: Total cholesterol
HDL: High-density lipoprotein
LDL: Low-density lipoprotein
TG: Triglycerides
EDTA: Ethylene diamine tetra acetic acid
CD: Cluster of differentiation
PBS: Phosphate-buffered saline
NHST: Null hypothesis significance testing
ANOVA: Analysis of variance
ANCOVA: Analysis of covariance
TSC: Trial Steering Committee
